# Alcohols react with MCM-41 at room temperature and chemically modify mesoporous silica

**DOI:** 10.1038/s41598-017-10090-x

**Published:** 2017-08-30

**Authors:** Sebastian Björklund, Vitaly Kocherbitov

**Affiliations:** 1 Department of Biomedical Science, Faculty of Health and Society, Malmö University, Malmö, Sweden; 2 Biofilms Research Center for Biointerfaces, Malmö University, Malmö, Sweden

## Abstract

Mesoporous silica has received much attention due to its well-defined structural order, high surface area, and tunable pore diameter. To successfully employ mesoporous silica for nanotechnology applications it is important to consider how it is influenced by solvent molecules due to the fact that most preparation procedures involve treatment in various solvents. In the present work we contribute to this important topic with new results on how MCM-41 is affected by a simple treatment in alcohol at room temperature. The effects of alcohol treatment are characterized by TGA, FTIR, and sorption calorimetry. The results are clear and show that treatment of MCM-41 in methanol, ethanol, propanol, butanol, pentanol, or octanol at room temperature introduces alkoxy groups that are covalently bound to the silica surface. It is shown that alcohol treated MCM-41 becomes more hydrophobic and that this effect is sequentially more prominent going from methanol to octanol. Chemical formation of alkoxy groups onto MCM-41 occurs both for calcined and hydroxylated MCM-41 and the alkoxy groups are hydrolytically unstable and can be replaced by silanol groups after exposure to water. The results are highly relevant for mesoporous silica applications that involve contact or treatment in protic solvents, which is very common.

## Introduction

Silica with ordered and accessible mesopores, such as MCM-41^1^ and SBA-15^2^, were successfully synthesized in the early 1990s by utilizing the principle of self-assembly of surfactants as structure-directing agents. Since then, mesoporous silica materials have received much attention in the field of nanotechnology due to their remarkable features of well-defined structural order, high surface area, narrow pore size distribution, and perhaps most important – its tunable pore diameter and surface properties^3^. These attractive properties have shown great promise in applications such as catalysis, adsorption, separation, sensing, drug delivery, and biosensors^4, 5^.

To employ mesoporous silica for applications like these it is important to design their structural features and surface properties in an optimized manner for the application in mind. The particle size, morphology, pore size, and pore structure can be rationally designed based on the knowledge of surfactant self-assembly and the chemistry of silica synthesis^6^. Once the mesoporous silica material is synthesized it is possible to change the chemical properties of the surface by post-synthesis modification, which can be performed according to several strategies.

In principle, most strategies for surface modification of mesoporous silica is possible due to the presence of surface silanol (Si-OH) groups, which exists either as isolated single or geminal silanols^7^. The Si-OH groups constitute the anchor onto which organic molecules can covalently bind by post-synthesis modification steps, such as silylation^8^. This is one example of why it is important to characterize the silanol chemistry of mesoporous materials to completely understand and control the surface modification process. The silanol number, n_OH_, which is defined as the number of Si-OH groups per unit surface area, has been a subject for much research and discussion with the main conclusion being that n_OH_ is subject to variation depending on the type and history of the silica material^9^. For example, exposure to humid air provides water molecules that react chemically with siloxane (Si-O-Si) groups to form Si-OH groups^10^ – a process referred to as hydroxylation. Hydroxylation is thus important to consider if the modification process depends on the initial degree of silanol groups that are available for chemically grafting of organic molecules. Further, increasing the number of Si-OH groups by hydroxylation makes the silica surface more hydrophilic and may therefore change how the material interacts with, for example, drug molecules during loading into the mesopores for controlled release applications or adsorption of polar molecules^7^.

A related and important topic is how mesoporous silica interacts with solvent molecules due to the fact that most applications in some step or another involve treatment of the mesoporous material in aprotic or protic solvents. For example, for controlled release applications the active ingredient is usually loaded into the mesopores by dissolving the drug in a solvent, together with the silica material, after which the solvent is evaporated to obtain drug loaded mesoporous silica^11^. In general, the excess reagents and/or byproducts of functionalization are removed by washing silica with solvents, such as toluene, methanol, water, aqueous solution with weak bases, etc. However, when the surface chemistry is scrutinized after such procedures significant concentrations of unwanted alkoxy species have been observed by solid-state^1^H and^13^C NMR analysis^12^. Thus, in many cases the influence of this type of treatments on the properties of the mesoporous silica is largely overlooked, in spite of the fact that the surface chemistry may change significantly.

One illustration of this issue is formation of Si-OR groups at the silica surface during treatment in alcohols at elevated temperatures, which has been reported previously^9, 13^. During this treatment alkoxy groups covalently bind to the silica surface and this modification has been commercially utilized for chromatographic supports (e.g. Durapak). However, since this material is hydrolytically unstable, as compared to Si-O-Si and Si-C linked phases, this chromatographic support is only relevant for non-aqueous mobile phases^14^.

With this as background, in the present study we extend this topic by investigating how MCM-41 is affected by a simple treatment in alcohol solvent at room temperature, which is a very common practice in many cases in synthesis and preparation procedures of mesoporous silica. The MCM-41 material was synthesized according to standard procedures and then dispersed in methanol, ethanol, propanol, butanol, pentanol, or octanol. The effects of this simple treatment were then characterized by TGA, FTIR, and water and hexane sorption calorimetry. The results are clear and show that alcohol treatment at room temperature introduces alkoxy groups that are covalently bound to the silica surface of MCM-41. The density of alkoxy groups after alcohol treatment is similar for both calcined silica and hydroxylated silica, suggesting that alcohols react with both siloxane and silanol groups to form alkoxy groups. The alkoxy groups are hydrolytically unstable and can be replaced by silanol groups after treatment in liquid water or water vapor. As observed by water sorption calorimetry, the chemically bonded alkoxy groups results in a clear hydrophobization of the silica surface and this effect is successively enhanced as the length of the carbon chain of the alkoxy group increases. The hexane sorption isotherms are in good agreement with the water sorption isotherms; both showing that the available pore volume of the alcohol treated MCM-41 is continuously decreased as the length of the alcohol carbon chain increases. The presented results are highly relevant for applications that are based on silica or mesoporous silica materials and involve any contact or (pre)treatment in protic solvents, which is a very common combination.

## Materials and Methods

### Materials

Cetyl trimethyl ammonium bromide (CTAB), ammonia, tetraethylorthosilicate (TEOS), 1-propanol (≥99%), 1-butanol (≥99%), 1-pentanol (≥99%), 1-octanol (≥99%), hexane (≥99%), benzene (≥99%), cyclohexane (≥99%), tert-butanol (≥99%) were purchased from Sigma-Aldrich. Methanol (anhydrous, 99.9%) was obtained from Alfa Aesar. Ethanol (anhydrous, 99.9%) was purchased from VWR.

### Synthesis of MCM-41

MCM-41 was synthesized according to previously published protocols^15^. In brief, 2.4 g CTAB was dissolved in 120 ml water at 30 °C and under constant mixing at 200 rpm with a magnetic stirrer. Next, 9.8 ml 25% ammonia solution and 10.72 ml TEOS was added. After 60 h the temperature was changed to 90 °C and kept for 24 h under continuous stirring to produce the MCM-41. After this step, the material was filtered (Munktell, 00 A) and washed several times with water before calcination. The calcination was performed by ramping the temperature at a rate of 1 °C/min from 25 to 550 °C, where it was kept at isothermal conditions for 5 h, after which the temperature was lowered again to 25 °C at a cooling rate of 5 °C/min.

### Modification of MCM-41 by alcohol treatment

After synthesis, the MCM-41 was dispersed in alcohol (approximately 20 mg in 1 ml alcohol) under reduced pressure to ensure that no air was trapped in the mesopores. During this process, the alcohol was evaporated in a time span of hours in the case of methanol, ethanol, and propanol. This time span was extended for butanol, while pentanol and octanol required an elevated temperature (approximately 80, and 150 °C, respectively), which in combination with reduced pressure was sufficient to remove these higher alcohols to obtain MCM-41 in powder form.

### SAXD

Small angle X-ray diffraction (SAXD) measurements were conducted on untreated MCM-41 with a compact Kratky camera from HECUS X-ray systems that is operating with a line collimated beam from a Cu source with K-alpha emission at λ = 1.542 Å.

### SEM

The morphology of untreated MCM-41 was examined with scanning electron microscopy (SEM, Zeiss EVO LS10) equipped with a LaB6 filament. MCM-41 was sputter-coated with gold using an Agar automatic sputter coater at 30 mA and 0.08 mbar of pressure for one min prior to analysis. The material was then imaged in high-vacuum mode using a secondary electron detector, at a 10 kV accelerating voltage, 50 pA probe current, and 9 mm working distance.

### Thermal gravimetric analysis (TGA)

TGA (Q500 TA Instruments) measurements were performed on MCM-41 samples treated in alcohols by ramping the temperature at a rate of 10 °C/min from 25 to 800 °C. The error of the TGA measurements was determined by analyzing the noise of the TGA data and was in all cases below ± 0.01 mg, which corresponds to an error below 0.5% for the smallest sample used in this work (sample masses ranged from approximately 5–10 mg, see Table S1). The TGA data are presented as the percentage of (m−m_final_)/m_final_, where m and m_final_ is the mass and final mass, respectively. By normalizing the data in this manner the TGA curves reflect the organic content (alcohol content) of the MCM-41 samples in a clear way.

### FTIR

Fourier transformed infrared (FTIR) spectra were obtained using a Thermo Nicolet Nexus 6700 instrument (Thermo Scientific). All measurements were performed on small pellets of MCM-41 (approximately 5 mg) by collecting 16 scans with a spectral range between 600–4000 cm^−1^ with a resolution of 0.4821 cm^−1^ (the background was collected under identical settings).

### Water and hexane sorption calorimetry

Sorption calorimetry is a method where one can simultaneously determine the sorption isotherm and the enthalpy of sorption. In practice, the technique is based on isothermal calorimetry and involves two calorimetric chambers that are placed on top of each other^16, 17^. The initially dry sample is placed in the top calorimetric cell, while the bottom calorimetric cell is empty from the start and later filled with pure solvent (water or hexane) by injection. The two cells are connected via a tube through which water or hexane vapor can diffuse from the bottom to the top cell. From the thermal power of vaporization, measured in the bottom cell containing the pure solvent, one can obtain the sorption isotherm. In addition, by combining the thermal powers measured in both cells one can calculate the partial molar enthalpy of mixing as a function of solvent content. The details of this technique is described elsewhere^10, 16, 17^. The MCM-41 samples, approximately 25 mg, were dried in presence of molecular sieves in a small glass tube at vacuum for a minimum time of 12 h. Finally, before starting the experiment the sample was loaded into the calorimetric cell under dry conditions by using a glove box filled with nitrogen gas.

## Results and Discussion

### Characterization of untreated MCM-41

The untreated MCM-41 material was characterized by SEM, SAXD, and water sorption calorimetry measurements. The SEM images show the typical morphology of MCM-41 and the SAXD data display a characteristic pattern corresponding to a hexagonal pore arrangement with peaks from the (100), (110), (200), and (210) reflections (see Fig. S1). The parameter a_0_, which represents the center-to-center distance (i.e. pore diameter plus wall thickness), was determined to 4.8 nm based on the (100) reflection at q = 1.51 nm^−1^.

**Figure 1:**
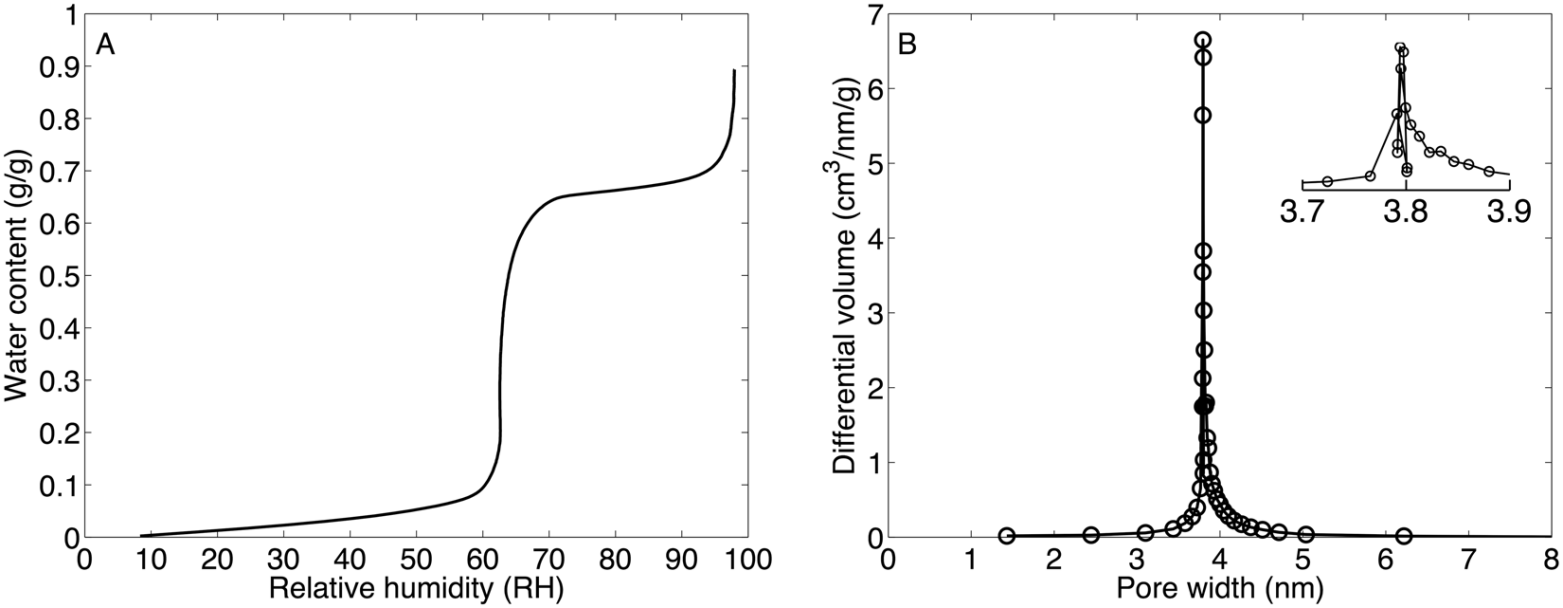
(**A**) Water sorption isotherm of untreated MCM-41. (**B**) Pore size distribution of untreated MCM-41 calculated with the BJH equation with the data in (A) (input parameters: θ = 34°, t = 0.24 Å^18, 19^). The inset in (**B**) shows a close up of the pore size distribution around 3.7–3.9 nm.

From the water sorption isotherm presented in Fig. 1A it is possible to calculate the pore size distribution (PSD) from the BJH (Barret–Joyner–Halenda) equation^18^ by the methodology described in detail elsewhere^19^. In these calculations, the values of the contact angle (θ = 34°) and thickness of the adsorbed water layer (t = 0.24 Å) were taken from previous studies of MCM-41^19^. From the BJH analysis, the pore size of the MCM-41 was determined to 3.8 nm (see Fig. 1B), which is in agreement with literature data^10, 19^. From the water sorption isotherm the volume of the mesopores was estimated from the end of the capillary condensation regime where complete filling of the pores is established. In this work we define this regime by the local maximum of the first derivative of the change of the relative humidity (see Fig. S2A). According to this analysis, the water content after capillary condensation was determined to 0.66 g/g. The apparent water density in MCM-41 has been established to 0.88 g/cm^3^ ^10^, which gives a total pore volume of V = 0.75 cm^3^/g ( = 0.66/0.88). If it assumed that the pores are cylindrical it is possible to calculate the pore length h from the volume V and the radius of the pores r according to h = V/π/r^2^. Next, the surface area A can be determined from geometrical considerations according to A = 2πrh = 2 V/r. From this analysis the surface area of untreated MCM-41 was determined to 786 m^2^/g, which is in satisfactory agreement with reported values on MCM-41 synthesized according to similar protocols as in this work (usually close to 1000 m^2^/g based on BET analysis of N_2_ sorption data)^10^. One reason for that the calculated surface area is lower than 1000 m^2^/g can be related to that the calculation assumes that the pores are cylindrical, which may lead to underestimation of the area. However, this does not influence any of the conclusions from the present study.

### Alcohol treated MCM-41

#### TGA

**Figure 2:**
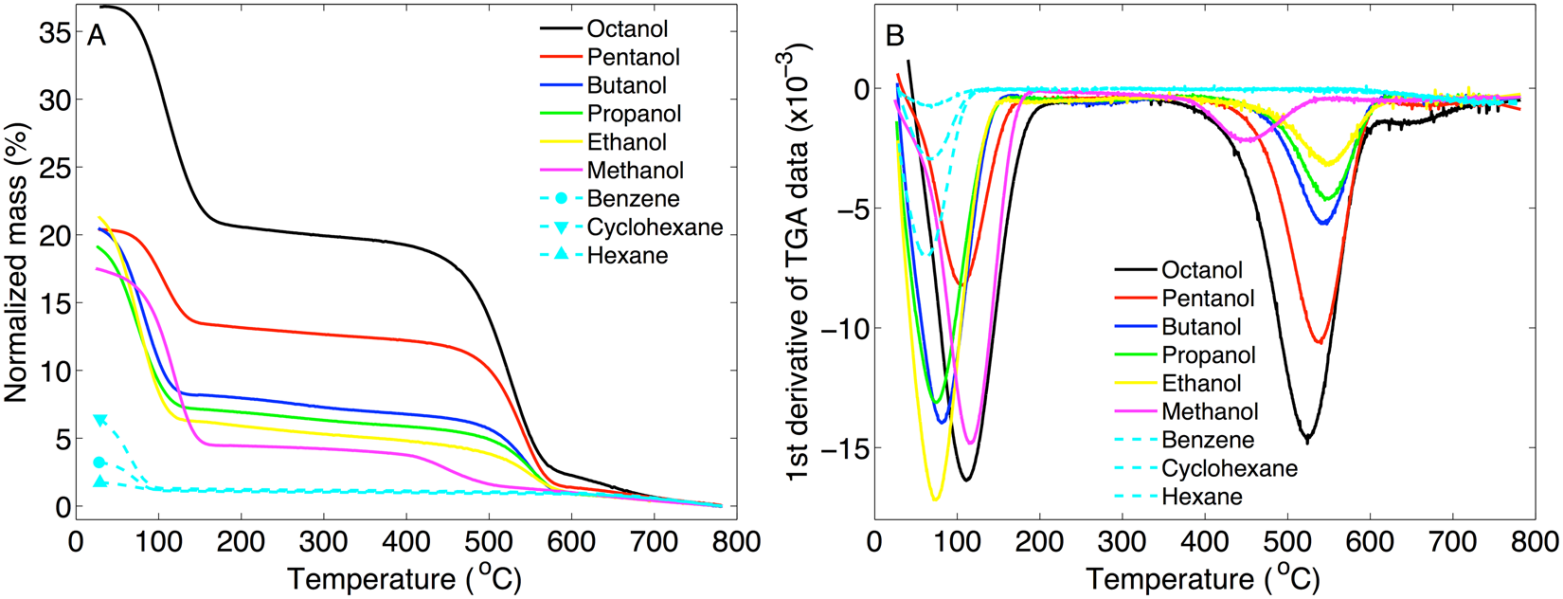
TGA results of MCM-41 treated in alcohols and some aprotic solvents. (**A**) Normalized mass as a function of temperature. (**B**) First derivative of the TGA curves in (**A**) as a function of temperature.

After treatment in alcohols the MCM-41 material was analyzed with TGA to investigate the alcohol content from the thermal gravimetric mass loss. The results are presented in Fig. 2A with the normalized mass being defined as (m − m_final_)/m_final_ (%) where m is the mass of the MCM-41 sample and m_final_ is the mass at the end of the TGA experiment. Thus, the normalized mass reflects the organic content of the MCM-41 sample.

The results in Fig. 2A show two clear mass loss steps, referred to as T_1,TGA_ and T_2,TGA_, between approximately T_1,TGA_ = 50–150 °C and T_2,TGA_ = 400–600 °C. Table S1 provides a compilation of the TGA data. The first step coincides with vaporization of pure alcohol (see Fig. S3) and therefore indicates vaporization of physically adsorbed alcohol from the MCM-41 mesopores.

The first derivative of the normalized mass is presented in Fig. 2B and shows that the maximal rate of mass loss occurs at similar temperatures, below 200 °C, for all alcohols and aprotic solvents.

The second step in the TGA curves occurs at much higher temperatures as compared to the boiling points of the alcohols and is therefore not related to vaporization of physically sorbed molecules. Thus, the second step (T_2,TGA_ = 400–600 °C) can only be attributed to mass loss of chemically bound molecules. To verify that the second step in the TGA curves is related to vaporization of molecules that are chemically bound to the silica via the alcohol functional group (i.e. Si-OR), and not via hydrocarbon atoms (i.e. Si-R), we performed control experiments where the MCM-41 samples were treated in the aprotic solvents cyclohexane, benzene, or hexane. As seen in Fig. 2 these molecules are completely vaporized between approximately 50–100 °C. This shows that aprotic solvents are not chemically bound to MCM-41, while the alcohols are likely bound to the silica in the form of alkoxy groups (i.e. according to Si-OR). The results also imply that the mass loss at T_2,TGA_ cannot be due to trapped alcohol molecules within the mesoporous network as this mechanism is not likely to discriminate between alcohols or aprotic molecules (or water molecules). To verify this suggestion we performed additional control experiments. In the first control experiment the MCM-41 material was treated in tert-butanol, instead of normal butanol (see Fig. S4A). The rationale behind this control experiment is that tert-butanol is expected to be less reactive with the MCM-41 material, as compered to normal butanol, due to sterical hindrance and that the -OH group of tert-butanol is interacting in a hydrogen-bonding network (both in bulk liquid state and in nanoporous confinement)^20^. However, the less reactive nature of tert-butanol would not prevent this molecule from diffusing into the porous network of MCM-41, which in principle could result in trapped, but not covalently bound, tert-butanol molecules. As seen in Fig. S4A the second mass loss step between 400–600 °C is not observed in the TGA curve corresponding to the MCM-41 sample treated in tert-butanol. In the second control experiment the octanol treated MCM-41 sample was thoroughly washed in hexane under mixing at elevated temperature of 55 °C for 1 h. After this procedure the sample was centrifuged after which the supernatant was exchanged with fresh hexane. This cycle was repeated 4 times in total before the washed MCM-41 sample was investigated by TGA (see Fig. S5A). From the results it is clear that the mass loss at T_2,TGA_ is still present after this washing procedure, while the mass loss from the first step at T_1,TGA_ is absent. Taken together, the mass loss at T_2,TGA_ can convincingly be attributed to chemically attached alkoxy groups, while the mass loss at T_1,TGA_ is due to alcohol molecules that are physically absorbed (trapped) in the mesoporous network. The latter fraction can thus be removed by rigorous washing in a suitable solvent, such as hexane.

#### FTIR

Information on the molecular scale was obtained from FTIR experiments on alcohol treated MCM-41. In these experiments the MCM-41 samples were investigated in the following three steps:

1. After treatment in alcohol.
2. After being heated to 350 °C (i.e. above T_1,TGA_ and below T_2,TGA_, see Fig. 2).
3. After being heated to 800 °C (i.e. above T_2,TGA_, see Fig. 2).

**Figure 3:**
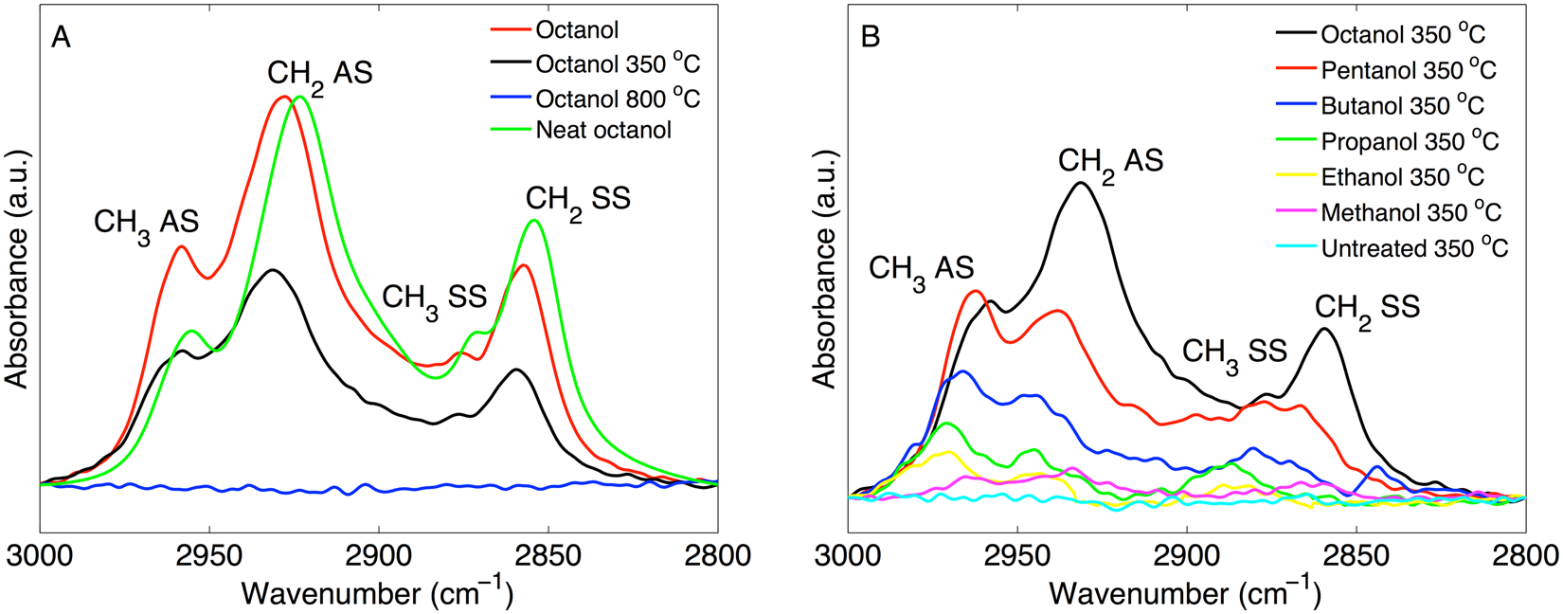
Hydrocarbon (CH) vibrations of MCM-41 treated in alcohols. (**A**) MCM-41 treated in octanol. (**B**) MCM-41 treated in alcohols and then heated to 350 °C. Abbreviations: CH_3_ AS – methyl antisymmetric stretch, CH_3_ SS – methyl symmetric stretch, CH_2_ AS – methylene antisymmetric stretch, CH_2_ SS – methylene symmetric stretch. The intensity of all spectra are normalized with respect to the most prominent absorption band between 950–1250 (not shown here), which is assigned to antisymmetric stretching of siloxane^21^. However, the intensity of the spectrum corresponding to neat octanol (green spectrum in A) is arbitrarily scaled to allow for comparison.

Representative results from the FTIR experiments are presented in Fig. 3A for octanol treated MCM-41, while Fig. 3B shows spectra of MCM-41 treated in the different alcohols and then heated to 350 °C.

The results in Fig. 3A clearly show that octanol is present in the MCM-41 material at room temperature (red spectrum). Further, the results show clear peaks of alkyl chain vibrations in the spectrum corresponding to the sample that was heated to 350 °C, while the spectrum from the sample that was heated to 800 °C does not show any signs of alkyl vibrations. The results in Fig. 3A are general for all MCM-41 samples that were treated in different alcohols and are in line with the mass loss steps in the TGA curves.

The results in Fig. 3B demonstrate that all spectra, from the different alcohol treated MCM-41 samples, contain peaks from methyl and methylene groups after being heated up to 350 °C. A general feature of the spectra is that the intensity of the peaks from CH vibrations is sequentially more prominent as the number of hydrocarbons of the alcohol used to treat the MCM-41 sample increases. In contrast to these results, it should be noted that the FTIR spectra from the MCM-41 sample treated in tert-butanol did not show any signs of C-H vibrations after heated to 350 °C (see Fig. S4B). In addition, it should be noted that the FTIR spectrum from the octanol treated MCM-41 sample that was thoroughly washed in hexane at 55 °C showed clear peaks from C-H vibrations (see Fig. S5B).

#### Combined results from TGA and FTIR

**Figure 4:**
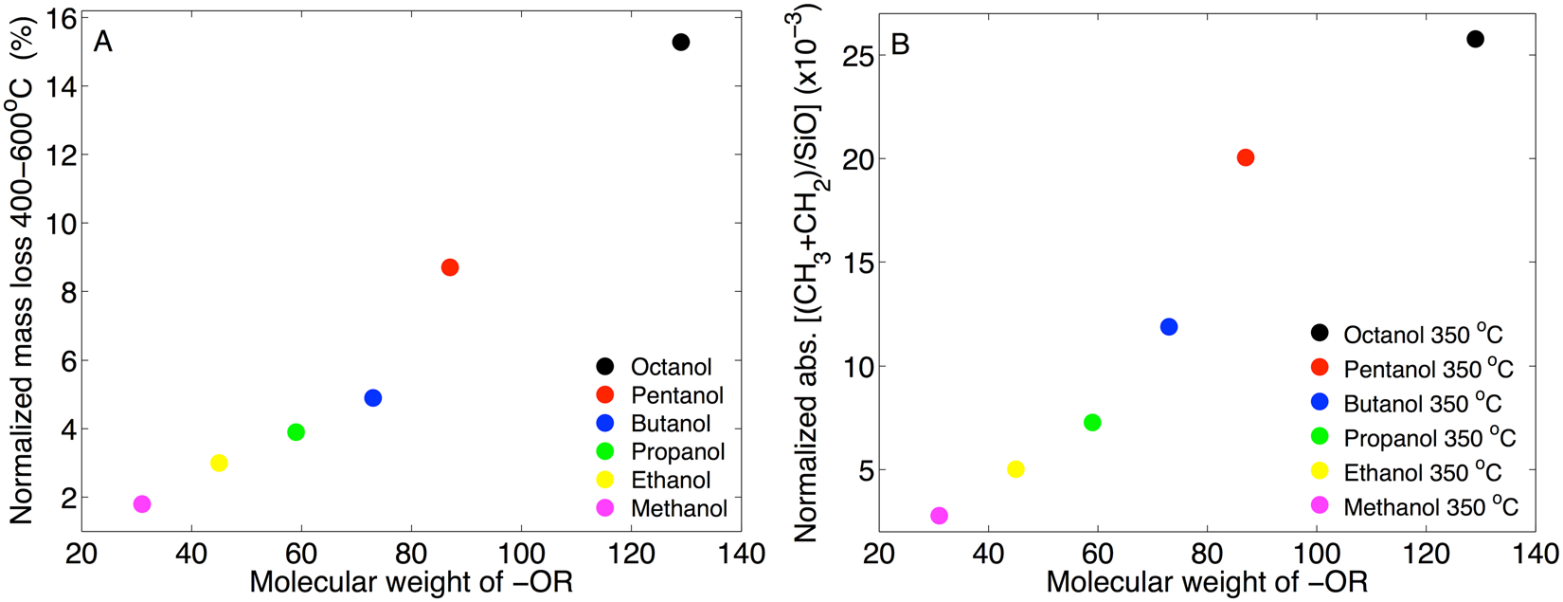
Summary of (**A**) TGA and (**B**) FTIR results. In (**A**) the mass loss between 400 °C and 600 °C was calculated after baseline correction (see text below). In (**B**) the absorbance was determined by deconvolution of the peaks from CH_3_ antisymmetric stretch and CH_2_ antisymmetric stretch vibrations (see Fig. 3B) by using a mix of Lorentzian and Gaussian line shapes. Next, the absorbance from CH was summed up and normalized with respect to the maximal absorbance from antisymmetric stretching of siloxane (Si-O) around 1050 cm^−1^.

Based on the TGA data we can conclude that the mass of the alkoxy groups covalently bound to the MCM-41 silica successively increases as a function of molecular weight of the alcohol used to treat the MCM-41 sample at room temperature. Similarly, from the FTIR results it can be concluded that the number of CH_2_ and CH_3_ molecular segments that are chemically grafted to the MCM-41 samples after heating to 350 °C increases in a sequence going from treatment in methanol to octanol. For clarity, the complementarity of these results are summarized in Fig. 4A and B for TGA and FTIR, respectively.

#### Water and hexane sorption calorimetry

**Figure 5:**
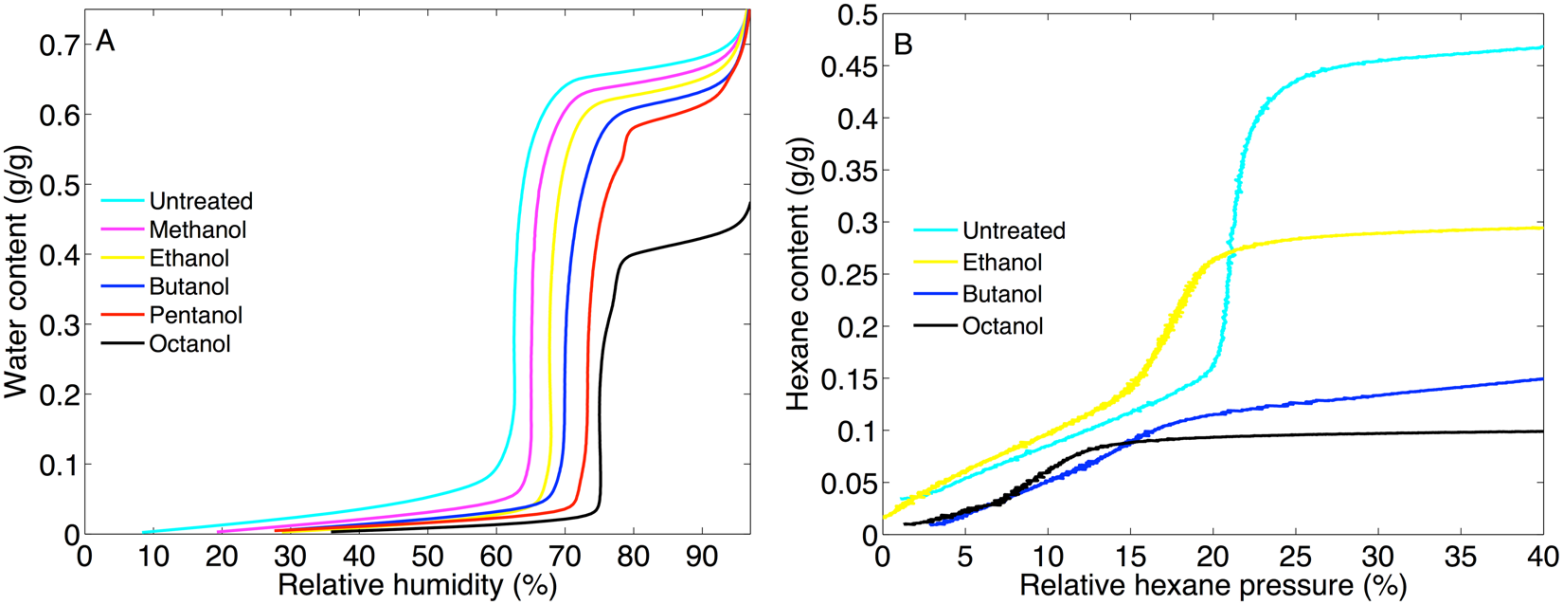
(**A**) Water sorption isotherms of MCM-41 and alcohol treated MCM-41 showing the water content (g water/g dry MCM-41 sample) as a function of relative humidity (%). (**B**) Hexane sorption isotherms of MCM-41 and ethanol, butanol, and octanol treated MCM-41 showing the hexane content (g hexane/g dry MCM-41 sample) as a function of relative hexane pressure (%). It should be noted that the alcohol treated MCM-41 samples were dried in vacuum with molecular sieves for 12 h minimum before the sorption calorimetry experiments to minimize presence of excess of alcohols in the MCM-41 material.

The water and hexane sorption isotherms were determined by performing sorption calorimetry experiments. The results from these experiments are shown in Fig. 5A and B, respectively.

The results in Fig. 5A show that the water sorption isotherms of the MCM-41 samples are strongly influenced by the alcohol treatment and several conclusions can be drawn based on the results. Firstly, at low humidities (i.e. below capillary condensation) the alcohol treated MCM-41 samples take up less water as compared to untreated MCM-41. This is clearly seen by comparing the water content at a specific relative humidity below the capillary condensation humidity, for example at RH = 50%. Secondly, the humidity required to induce capillary condensation of water in the mesopores is successively shifted to higher RH values. Based on the Kelvin-Cohan equation, this shift can only be explained by either that the size of the mesopores increases after treatment in alcohol, which is highly unexpected, or that the silica surface becomes more hydrophobic resulting in an increased contact angle^19^. Again, it is unlikely that the size of the mesopores increases after treatment in alcohol. This suggestion was confirmed by control experiments with SAXD showing that the unit cell dimensions of MCM-41, after treatment in alcohol, remain intact (see Fig. S1). In other words, the solid structure of the MCM-41 mesopores remains unchanged, while it is likely that a layer of chemically bound alkoxy groups is formed on the silica surface that results in successively increased hydrophobicity after treatment in higher alcohols.

The water sorption experiments were complemented with hexane sorption studies, which are presented in Fig. 5B showing good agreement with the water sorption isotherms. The first observation from the hexane isotherms is that the capillary condensation occurs at successively lower relative pressures, which implies that the pore volume is reduced after treatment in alcohols. For clarity the first derivative of the change of the relative hexane pressure is presented in Fig. S2B showing that the hexane content after capillary condensation is lowered from 0.46 g/g for untreated MCM-41 to 0.29, 0.14, and 0.10 g/g for MCM-41 treated in ethanol, butanol, and octanol, respectively. The second conclusion is that the hexane content after capillary condensation is continuously decreased after treatment in higher alcohols, which shows that the volume of the pores decreases successively after treatment in alcohols with longer carbon chains. This is consistent with the water sorption data.

The pore size distributions, corresponding to the hexane isotherms in Fig. 5B, were calculated based on the BJH equation following the same procedures as described above for water sorption (see ref. 19 for details) were the contact angle was set to zero (θ = 0°) and t = 5 Å^22^. The PSDs are presented in Fig. S6A. The pore widths for the alcohol treated samples were sequentially reduced from 3.2 nm for untreated MCM-41 to 2.9, 2.5, and 2.0 for MCM-41 treated in ethanol, butanol, and octanol, respectively (see Fig. S6A). This confirms that the shift in relative humidity required to induce capillary condensation of water in the mesopores (see Fig. 5A) can only be explained by an increase of hydrophobicity of the silica surface resulting in an increased contact angle.

Taken together, the hexane sorption data (see Table S2) are in good agreement with previous studies on folded sheet mesoporous material (FSM-16) showing similar reduction of the pore volumes, pore widths, and surface areas after treatment in ethanol, butanol, and octanol at elevated temperature^13^.

The hydrophobic nature of the alcohol treated MCM-41 samples is confirmed by the hydration enthalpies for the different MCM-41 samples (see Fig. S6B). The hydration enthalpy for the untreated MCM-41 sample indicates that the initial hydration is exothermic, while the opposite is observed for the alcohol treated samples (i.e. endothermic initial hydration). This implies that water molecules interact favorably with the silanol groups of the untreated MCM-41 surface^10^, while the presence of alkoxy groups in the alcohol treated MCM-41 samples results in less favorable interactions.

#### Alcohols react chemically with MCM-41 at room temperature

The combination of the TGA, FTIR, and water and hexane sorption calorimetry data strongly supports the conclusion that a fraction of alcohols is chemically attached to the silica surface after treatment in alcohols at room temperature. Starting with the TGA results (Fig. 2), the first mass loss step of the TGA curves, between approximately 50–150 °C, represents vaporization of liquid alcohol that is either adsorbed to the silica surface or absorbed in the mesopores. This fraction of physically sorbed alcohols is lost when the MCM-41 samples are heated above approximately 150 °C (and certainly when heated to 350 °C, see Fig. 2A). The removal of the physically sorbed alcohol fraction by heating to 350 °C results in a clear decrease in the intensity of the FTIR peaks from methyl and methylene C-H vibrations, as compared to the non-heated alcohol treated MCM-41 sample at room temperature (see Fig. 3A). However, the fact that the FTIR peaks from methyl and methylene C-H vibrations remain after heating to 350 °C (cf. Fig. 3) proves that a fraction of the alcohols is chemically attached to the MCM-41 sample. The fraction of covalently bound alcohols is not vaporized until approximately 400 °C where the second step in the TGA curves starts (see Fig. 2A). The FTIR results from the samples that were heated to 800 °C confirm that the second TGA step is due to covalently bound alkoxy groups as any signs of methyl or methylene C-H vibrations are totally absent in the spectra corresponding to these samples (Fig. 3A). The complementary results from TGA and FTIR in Fig. 4 clearly demonstrate that the mass of alkoxy groups that are covalently bound to the MCM-41 mesopores scales with the molecular weight of the alcohol used to treat the MCM-41 sample at room temperature. The effect of this is that the hydrophobicity of the MCM-41 surface increases, which is clearly illustrated by the water sorption isotherms in Fig. 5A showing that these samples are more hydrophobic as compared to the untreated MCM-41 sample and that this effect follows a sequential order going from methanol to octanol. This conclusion is confirmed by the hexane sorption results (Fig. 5B and Table S2) showing that the pore volume and the pore width of the MCM-41 mesopores decreases sequentially after treatment in ethanol, butanol, and octanol, respectively.

#### The grafting density of chemically bound alkoxy groups is similar for all alcohols

From the TGA data it is possible to estimate the number of chemically attached alkoxy groups (n_OR_) per nm^2^ of the MCM-41 sample from the following equation:

1 $${n}_{OR}=\frac{{m}_{TGA}{N}_{A}}{{M}_{w}A}$$

**Figure 6:**
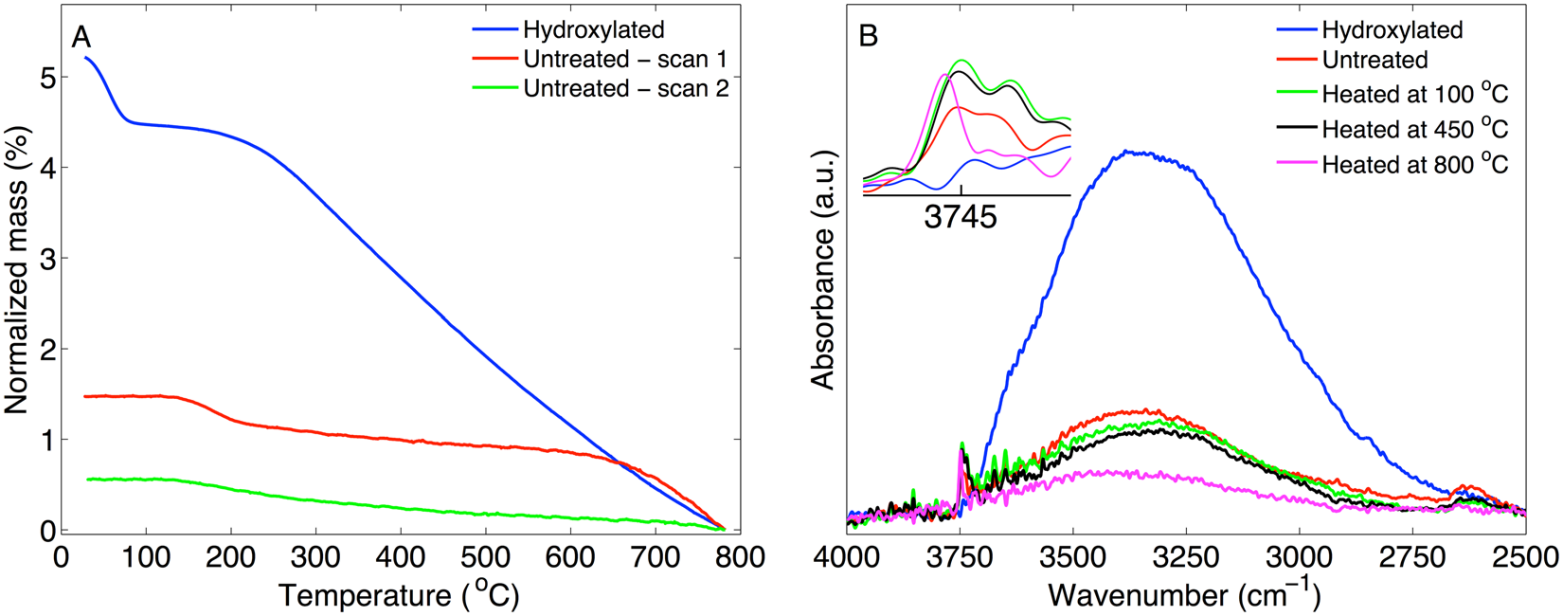
(**A**) TGA curves of hydroxylated MCM-41, untreated MCM-41, and freshly calcined MCM-41 (i.e. scan 2). (**B**) FTIR spectra of hydroxylated MCM-41, untreated MCM-41, and untreated MCM-41 heated to 100 °C, 450 °C, or 800 °C.

In Eq. 1, m_TGA_ (g g^−1^) is the mass that is lost due to vaporization of alkoxy groups, which occurs primarily between approximately 400 °C and 600 °C (i.e. the second step in the TGA curves), N_A_ is Avogadro’s number (mol^−1^), M_W_ (g mol^−1^) is the molecular weight of the alkoxy group, and A is the surface area (786 m^2^/g). It is important to underline that m_TGA_ only should reflect mass loss from alkoxy groups and that this parameter must not be influenced by vaporization of other material in general and hydroxyl groups in particular. To approach this problem we performed control experiments with TGA and FTIR. This set of control experiments were conducted with untreated MCM-41 and MCM-41 treated in water to introduce hydroxyl groups. The untreated MCM-41 sample was also investigated by a second TGA scan, recorded immediately after the first scan. The results from these experiments are presented in Fig. 6.

From the TGA results in Fig. 6A on hydroxylated MCM-41 it is clear that the slope of the TGA curve from the hydroxylated sample is different as compered to the curves from the untreated and the freshly calcined samples. This verifies that the MCM-41 is highly hydroxylated after treatment in water and that -OH groups are continuously vaporized during the TGA experiment. This observation is confirmed by the FTIR results in Fig. 6B where the spectrum from the hydroxylated sample shows a prominent broad band from hydrogen-bonded silanols, between approximately 3700–2800 cm^−1^, while the peak from free silanol groups at 3745 cm^−1^ is absent (see inset). In other words, the hydroxylated sample contains silanol groups that are connected via hydrogen bonds. Upon heating the sample, the band from the hydrogen-bonded silanols is successively attenuated, while the intensity of the peak corresponding to free silanols increases. Taken together, the number of silanol groups that are connected via hydrogen bonds are successively decreasing due to vaporization of -OH groups, which results in a relatively larger population of single and free silanol groups that are not interacting via hydrogen bonds.

**Figure 7:**
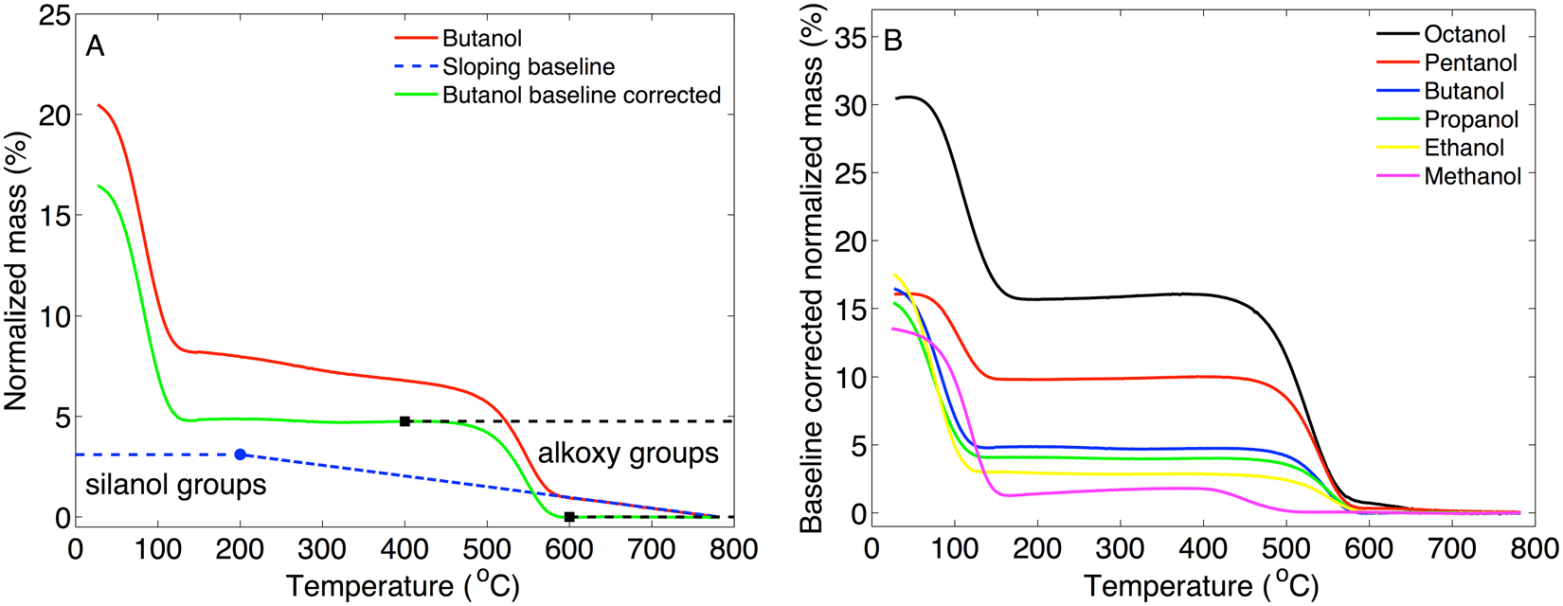
(**A**) Illustration of baseline correction. The correction was done by subtracting a sloping baseline, corresponding to the slope of the curves between approximately 700 °C and 800 °C. Next, the mass loss from the alkoxy groups was calculated from the step between approximately 400 and 600 °C (given by the mass loss between the black square markers). To estimate the mass loss due to vaporization of silanol groups, the baseline was extrapolated to 200 °C (given by the blue circle marker), where the onset of dehydroxylation from heating the sample occurs (see Fig. 6A). (**B**) Baseline corrected TGA curves from alcohol treated MCM-41.

Interestingly, the TGA curve from the hydroxylated MCM-41 sample shows an almost identical slope as the alcohol treated samples in the temperature interval where no clear mass loss step is observed (for example between 700–800 °C, see Fig. S7). This implies that the alcohol treated samples contain a significant number of hydroxyl groups that are continuously vaporized during the TGA experiments. Thus, to obtain a more reliable value of m_TGA_ in Eq. 1 it is required to correct for the mass loss due to silanol groups. This correction was systematically performed by subtracting a sloping baseline, corresponding to the slope of curve between approximately 700 °C and 800 °C, from the TGA curves where the mass loss from silanol groups is expected to dominate (see Fig. 7).

Effectively, the base line correction, illustrated in Fig. 7A, separates the mass loss due to vaporization of silanol groups from the mass loss due to vaporization of chemically attached alkoxy groups and allows for determination of both n_OR_ and n_OH_. The results from this analysis are compiled in Table 1. For comparison, it can be noted that the number of silanol groups for untreated MCM-41 (cf. Figure 6A) was determined to 1.2 ± 0.1 (n = 3) and 3.9 ± 0.3 (n = 4) for hydroxylated MCM-41. From the second TGA scan on untreated MCM-41 (cf. Figure 6A), the corresponding value of silanol groups was determined to n_OH_ = 0.5. In this comparison it is also relevant to include the silanol number of fully hydroxylated silica, which has been established to 4.6 ± 0.5 (based on 100 SiO_2_ samples)^23^. Thus, in the present study the MCM-41 sample was close to fully hydroxylated after treatment in water at room temperature. It should also be noted that the silanol numbers for untreated MCM-41 (i.e. not hydroxylated or treated in alcohol), which were calcined at 550 °C or heated to 800 °C in the TGA measurement, are in good agreement with literature data on n_OH_ as a function of pretreatment temperature^23^.

**Table 1:** Number of alkoxy n_OR_ and silanol n_OH_ groups per nm^2^ of alcohol treated MCM-41. Samples marked with * were exposed to elevated temperatures to enable evaporation of excess alcohol solvent after treatment (T ≈ 80 °C for MCM-41 treated in pentanol and T ≈ 150 °C for MCM-41 treated in octanol).

MCM-41 treatment	n_OR_	n_OH_	n_OR_ + n_OH_
Methanol (n = 2)	0.4 ± 0.1	2.8 ± 0.2	3.2 ± 0.3
Ethanol (n = 2)	0.5 ± 0.0	2.2 ± 0.4	2.7 ± 0.5
Propanol (n = 1)	0.5	2.5	3.0
Butanol (n = 2)	0.5 ± 0.1	2.4 ± 0.1	2.8 ± 0.2
*Pentanol (n = 2)	0.8 ± 0.0	3.8 ± 0.1	4.6 ± 0.2
*Octanol (n = 1)	0.9	3.5	4.4

The data in Table 1 show that the number of alkoxy groups is between 0.4–0.5 per nm^2^ for the MCM-41 samples treated in the lower alcohols and between 0.8–0.9 for pentanol and octanol. It is possible that the elevated temperatures used to remove these alcohols resulted in increased number of chemically attached alkoxy groups (see samples marked with * in Table 1). The corresponding silanol number is between 2.2–2.8 per nm^2^ for the samples treated in methanol, ethanol, propanol, and butanol; while the pentanol and octanol treated samples have higher silanol numbers between 3.5–3.8 per nm^2^.

The results in Table 1 indicate that the number of silanol and alkoxy groups that are introduced on the MCM-41 material during alcohol treatment at room temperature is connected. In other words, the formation of alkoxy and silanol seems to occur in a parallel manner with relatively higher numbers for pentanol and octanol treated MCM-41. This is clearly illustrated by the sum of n_OR_ + n_OH_ (see Table 1), which is between 2.7–3.2 nm^2^ for the samples treated in methanol, ethanol, propanol, and butanol and roughly 4.4–4.6 per nm^2^ for the pentanol and octanol treated samples. Notably, the sum of n_OR_ + n_OH_ corresponding to the MCM-41 samples treated in pentanol and octanol is similar to the silanol number of fully hydroxylated silica (i.e. 4.6 ± 0.5)^23^. Again, it is possible that these higher numbers for pentanol and octanol treated MCM-41 is related to the elevated temperature used to evaporate the excess alcohol after treatment resulting in saturation of hydroxyl and alkoxy groups on the silica surface. Chemical binding of alkoxy groups have previously been investigated for folded sheet mesoporous material (FSM-16) by refluxing the silica in alcohols for 24 h^13^. After this treatment, involving elevated temperature at 150 °C, the number of alkoxy groups on the silica surface was determined to be 2.3, 1.8, and 1.5 per nm^2^ after treatment in ethanol, butanol, and octanol, respectively, based on combustion analysis combined with N_2_ adsorption measurements^13^. These numbers are in reasonable agreement with the present results considering that the procedure of prolonged refluxing at 150 °C is expected to result in more efficient reaction conditions with higher number of alkoxy groups formed on the silica surface.

The present results can be rationalized by referring to the chemical reactions given in Eqs. 2 and 3.

2 $$\mathrm{ROH}\,+\equiv \mathrm{Si}-O-\mathrm{Si}\equiv \,\rightleftharpoons \,\mathrm{RO}-\mathrm{Si}\equiv +\mathrm{HO}-\mathrm{Si}\equiv $$

3 $${\rm{R}}{\rm{O}}{\rm{H}}+{\rm{H}}{\rm{O}}-{\rm{S}}{\rm{i}}\equiv \,\rightleftharpoons \,{\rm{R}}{\rm{O}}-{\rm{S}}{\rm{i}}\equiv +{{\rm{H}}}_{2}{\rm{O}}$$

Eqs. 2 and 3 show possible reactions for grafting of alkoxy and silanol groups; the difference between them is that Eq. 2 results in formation of both alkoxy and silanol groups on the silica surface, while Eq. 3 only results in formation of alkoxy groups (and free water). The results in Table 1 indicate that both alkoxy and silanol groups are formed after treatment in alcohol according to Eq. 2. It should be noted that the number of silanol groups is in general higher as compared to untreated MCM-41 (n_OH_ = 1.2 ± 0.1), which further supports the suggestion that formation of alkoxy groups are associated with introduction of new silanol groups according to Eq. 2. In addition, Kimura *et al*. suggested that only silanol groups react to form Si-OR groups after refluxing mesoporous silica in alcohols^13^. However, to what extent Eq. 2 or Eq. 3 is dominating is likely dependent on the initial degree of silanol or siloxane groups of the MCM-41 sample. This is confirmed by a control experiment that we performed by treating a close to fully hydroxylated MCM-41 sample in alcohol (see Fig. S8). Even though the TGA curves are not identical, the main conclusion from this control experiment is that alcohols chemically react with hydroxylated MCM-41 at room temperature to form covalently bound alkoxy groups in a similar manner as non-hydroxylated MCM-41 (i.e. untreated MCM-41). Thus, these results confirms that both Eqs. 2 and 3 can occur and that the type of reaction scheme depends on the initial degree of silanol or siloxane groups of the MCM-41 sample.

#### Covalently bound alkoxy groups are hydrolyzed by water at room temperature

**Figure 8:**
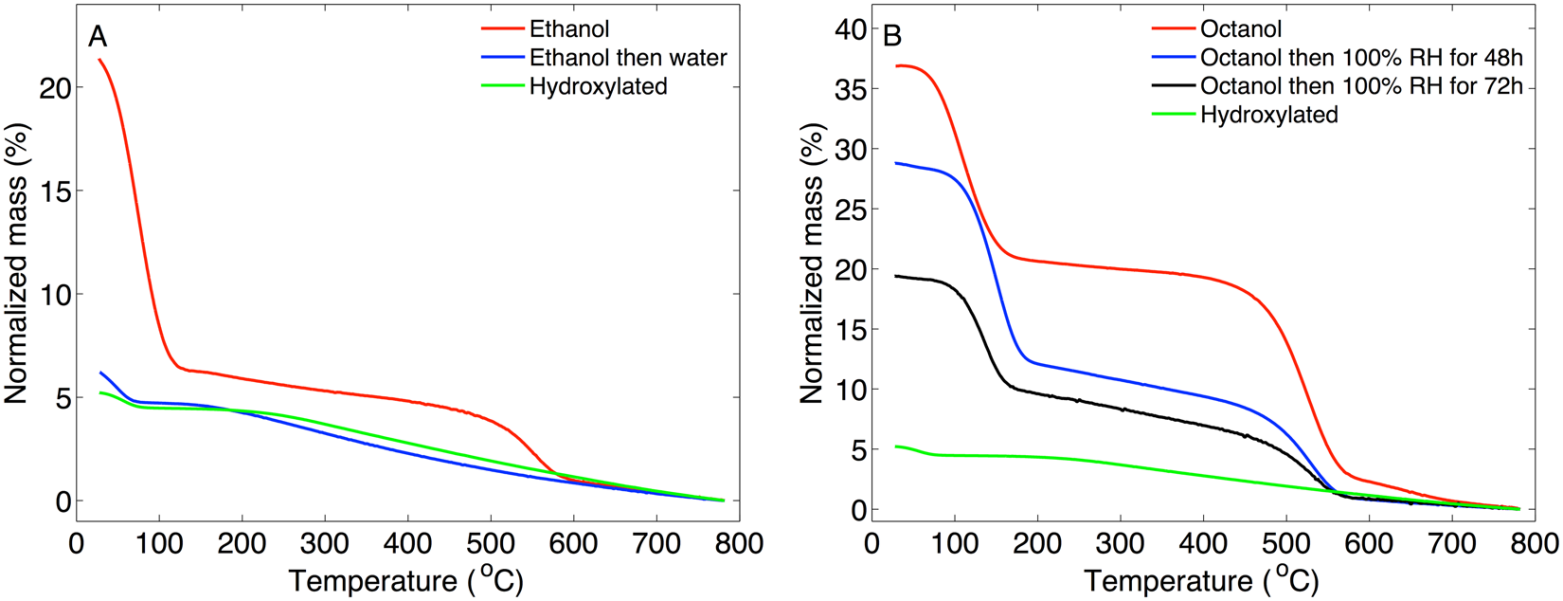
(**A**) TGA curves of ethanol treated MCM-41, before and after treatment in water. (**B**) TGA curves of octanol treated MCM-41, before and after treatment exposure to 100% relative humidity (RH) for 48 h, and 72 h. TGA curve for hydroxylated MCM-41 is included for comparison.

To investigate how the chemically bound alkoxy groups of alcohol treated MCM-41 were influenced by water we performed additional experiments were ethanol or octanol treated MCM-41 material was exposed to pure water or water vapor (the octanol treated MCM-41 was immiscible with liquid water why water vapor was used). The results from these experiments are shown in Fig. 8. For the ethanol treated MCM-41 sample the TGA curve has identical shape as compared to hydroxylated MCM-41. In other words, the covalently bound ethoxy groups are hydrolytically unstable and easily replaced by silanol groups after soaking the ethanol treated MCM-41 material in liquid water. Similarly, from FTIR measurements on the alcohol treated MCM-41 samples after water sorption calorimetry, during which the sample is exposed to water vapor for several days, it was concluded that the alkoxy groups were absent for the samples treated in methanol, ethanol, propanol, and butanol. However, for the MCM-41 samples treated in pentanol and octanol the FTIR spectra showed peaks from CH vibrations also after water sorption calorimetry. To further investigate this effect we exposed octanol treated MCM-41 to 100% RH for 48 h and 72 h after which TGA experiments were performed. The results are shown in Fig. 8B and show that the fraction of chemically attached octanol molecules is continuously reduced after prolonged exposure to 100% RH, as judged from the reduction of the second step between 400 and 600 °C in the TGA curves.

#### Covalently bound Si-OR groups may explain changes of dynamical properties of molecules inside mesoporous silica

Studies have observed that glass-forming liquids, such as glycerol or propanediol, can have either increased or decreased glass transition temperatures or dynamical properties, relative to bulk, when present inside the confined space of mesoporous silica^24, 25^. This complex behavior is likely dependent on several factors including type of mesoporous material, properties of the liquid molecules, and interactions between the silica and the glass-forming liquid. Thus, this complicated issue has not been unambiguously resolved yet.

Huwe *et al*. performed impedance spectroscopy studies on MCM-41 and MCM-48 material treated in 1,2-propanediol and observed that the dynamics of 1,2-propanediol was retarded when being present inside the mesopores, as compared to the bulk^25^. After silylation of the MCM-41 and MCM-48 material the relaxation rate of 1,2-propanediol was comparable to the bulk liquid^25^. Based on these observations, it was suggested that formation of hydrogen bonds between propanediol and the silica pore walls was the reason for slower dynamics and that silylation of the silica removed the possibility to form hydrogen bonds between silanol groups and propanediol^25^. Trofymluk *et al*. studied the dynamical properties of glycerol, among other molecules, inside the mesopores of MCM-41 and SBA-15 by differential scanning calorimetry and observed an increase of the glass transition temperature (reflecting slower dynamics), as compared to bulk glycerol. Unfortunately it was unclear whether this increase of glass transition temperature could be attributed to interactions between glycerol and surface silanols or to intermolecular interactions of the confined glycerol molecules, or both^24^. However, based on the results of the present study it is possible that this kind of molecules (e.g. 1,2-propanediol, glycerol, etc) can react with mesoporous silica to form covalently bound alkoxy groups and thus influence the dynamical properties and glass transition temperatures. Hopefully, the present results will stimulate further research to establish a more complete understanding of the complex behavior of glass-forming liquids inside the confined geometry of mesoporous silica.

## Conclusions

To successfully employ mesoporous silica for nanotechnology applications it is important to consider the fact that many solvents may influence the mesoporous silica material. In fact, this issue is very general due to the fact that most preparation procedures, in some step or another, involve treatment of the mesoporous material in protic solvents. In the present work we show that MCM-41 is strongly influenced by formation of covalently bound alkoxy groups after a simple alcohol treatment where the mesoporous material was dispersed in alcohol at room temperature. After this simple treatment the MCM-41 material was characterized in detail by TGA, FTIR, and water sorption calorimetry experiment. The results are highly relevant for applications that are based on silica or mesoporous silica materials and involve any contact or (pre)treatment in protic solvents, which is a very common combination.

Based on the clear results of this work the main conclusions are:

- Alcohol treatment at room temperature introduces alkoxy groups that are covalently bound to the silica surface of MCM-41.
- The TGA results show two clear mass loss steps between approximately 50–150 °C and 400–600 °C. The first step is due to evaporation of liquid alcohol from the MCM-41 mesopores. The second mass loss step between 400–600 °C is due to mass loss of chemically bound alkoxy groups.
- The TGA results are confirmed by FTIR results showing that the number of CH_2_ and CH_3_ molecular segments that are chemically grafted to the MCM-41 samples, after heating to 350 °C, increases in a sequence going from treatment in methanol to octanol.
- The TGA and FTIR results are supported by results from water and hexane sorption calorimetry, showing that the surface of MCM-41 is becoming more hydrophobic after being dispersed in alcohol at room temperature, due to covalently bound alkoxy groups on the silica surface, and that this effect is sequentially enhanced after treatment in methanol, ethanol, propanol, butanol, pentanol, and octanol.
- The number of alkoxy groups per nm^2^ is similar for MCM-41 treated in methanol, ethanol, propanol, and butanol (n_OR_ ≈ 0.5) and about twice this value for pentanol and octanol (n_OR_ ≈ 1.0). The higher n_OR_ number for pentanol and octanol treated MCM-41 can be explained by the higher temperature used to remove the excess alcohol solvent for these samples.
- The chemical reaction where alkoxy groups are grafted onto the MCM-41 material at room temperature occurs both for calcined and hydroxylated MCM-41, which allows us to conclude that alcohols react both with siloxane and silanol groups to form alkoxy groups.
- The alkoxy groups are hydrolytically unstable and can be replaced by silanol groups after treatment in liquid water or water vapor. This effect is more prominent for MCM-41 treated in lower alcohols (e.g. methanol, ethanol, propanol, butanol) as compared to MCM-41 treated in higher alcohols (e.g. pentanol and octanol).

## Electronic supplementary material

Supporting Information
