# Supplementary material for: Alcohols react with MCM-41 at room temperature and chemically modify mesoporous silica
Source: Sci Rep. 2017 Aug 30;7:9960. doi: 10.1038/s41598-017-10090-x (PMC5577207; doi:10.1038/s41598-017-10090-x)
Supplement: Supplementary file 1 — Supporting Information [file 41598_2017_10090_MOESM1_ESM.pdf]

Supporting Information

Alcohols react with MCM-41 at room temperature and chemically modify mesoporous silica

Sebastian Björklund[1,2,*] and Vitaly Kocherbitov[1,2]

[1]Department of Biomedical Science, Faculty of Health and Society, Malmö University, Malmö, Sweden

[2]Biofilms Research Center for Biointerfaces, Malmö University, Malmö, Sweden

[*]Corresponding author: Sebastian.bjorklund@mah.se

Table S1. Boiling points (T_{bp}) of pure alcohols and compilation of TGA data. Sample masses ranged from approximately 5-11 mg. The temperatures where the maximal rate of mass loss occurs during the first ($T_{1,TGA}$=50-150°C) and the second ($T_{2,TGA}$=400-600°C) step in the TGA curves in Fig. 2A in the main article (units in °C). The mass loss corresponding to $T_{1,TGA}$ and $T_{2,TGA}$ is included and given in units of percent (%). For comparison, the experimental results from MCM-41 treated in hexane, benzene, cyclohexane, and water are included. In general, $T_{1,TGA}$ occurs at successively elevated temperatures, which may be explained by the sequential higher boiling points for the different alcohols.

Treatment	T_{bp}	Sample mass (mg)	$T_{1,TGA}$	Mass loss $T_{1,TGA}$	$T_{2,TGA}$	Mass loss $T_{2,TGA}$
Methanol	65	9.4	115	13	450	4
Ethanol	78	4.5	73	15	550	5
Propanol	97	6.2	76	12	545	6
Butanol	118	5.6	82	12	541	7
Pentanol	138	8.9	104	7	537	12
Octanol	195	5.3	111	17	523	20
Hexane	68	5.3	68	1	-	-
Benzene	80	6.1	68	2	-	-
Cyclohexane	81	6.1	63	5	-	-
Water	100	11	57	1	-	-

Table S2. Water or hexane contents after capillary condensation (cap. cond.), pore volumes, surface areas, and pore widths of untreated MCM-41 and alcohol treated MCM-41. These parameters were determined according to the procedure described in the main article (see *Characterization of untreated MCM-41* in the main article and Figs. S2 and S6A). Values marked with * are from hexane sorption calorimetry and calculated with the following input parameters: surface tension γ = 18.0 mN/m, molar volume = 130.7e-6 m^3/mol, density ρ = 654 kg/m^3, θ=0°, t=5 Å. The fact that contact with water vapor leads to hydrolysis of the covalently bound alkoxy groups during the water sorption experiment means that this method is not appropriate to employ for determination of pore volume, pore width, and surface area for this kind of samples (only for the untreated MCM-41 sample). Therefore these data are excluded for the water sorption calorimetry experiments.

Treatment	Water / hexane content after cap. cond. (g/g)	Pore vol. (cm^3/g)	Pore width (nm)	Surface area (m^2/g)
Untreated	0.66 / 0.46*	0.75 / 0.70*	3.8 / 3.2*	786 / 879*
Methanol	0.64	-	-	-
Ethanol	0.62 / 0.29*	0.44*	2.9*	612*
Butanol	0.61 / 0.14*	0.21*	2.5*	343*
Pentanol	0.59	-	-	-
Octanol	0.40 / 0.10*	0.15*	2.0*	306*

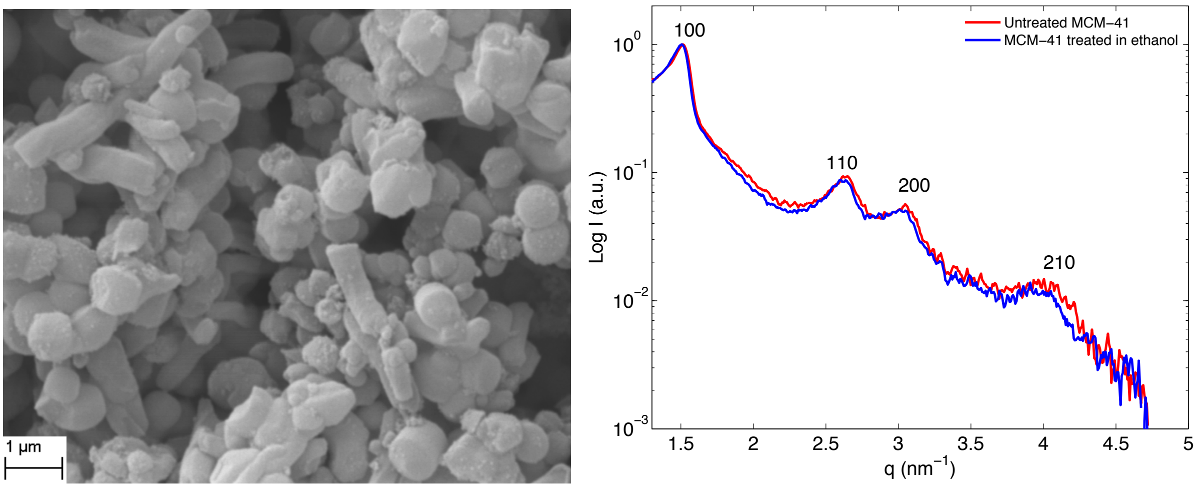

Figure S1. (Left) SEM image of untreated MCM-41. (Right) SAXS data of untreated MCM-41 and MCM-41 treated in ethanol.

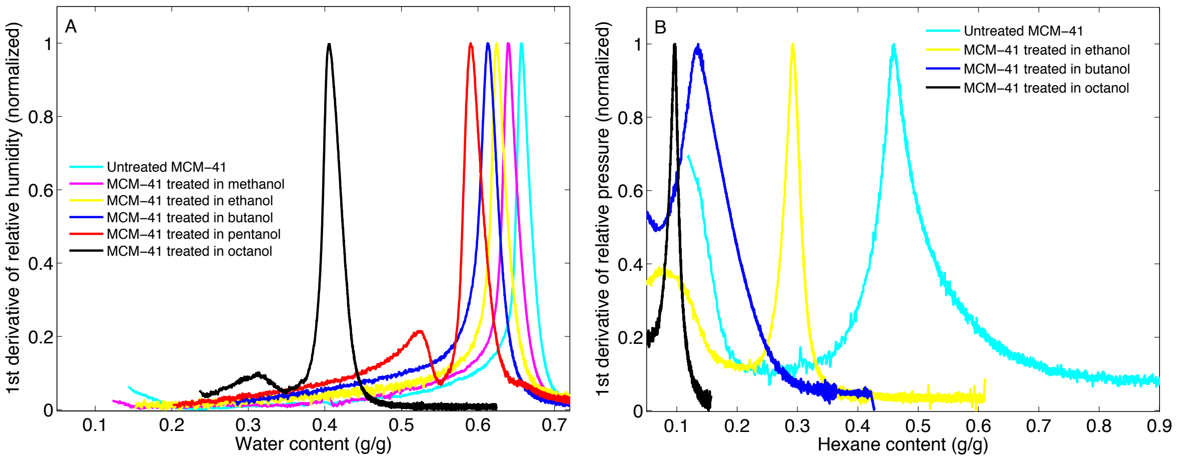

Figure S2. The 1st derivative of the relative humidity, or hexane pressure, as a function of water or hexane content in A and B, respectively. The corresponding sorption isotherms is presented in the main article in Fig. 1A and Fig. 5A for water and in Fig. 5B for hexane. The local maximum of the 1st derivative of the relative pressure, which corresponds to the water or hexane content after capillary condensation, occurs at sequentially lower water or hexane contents.

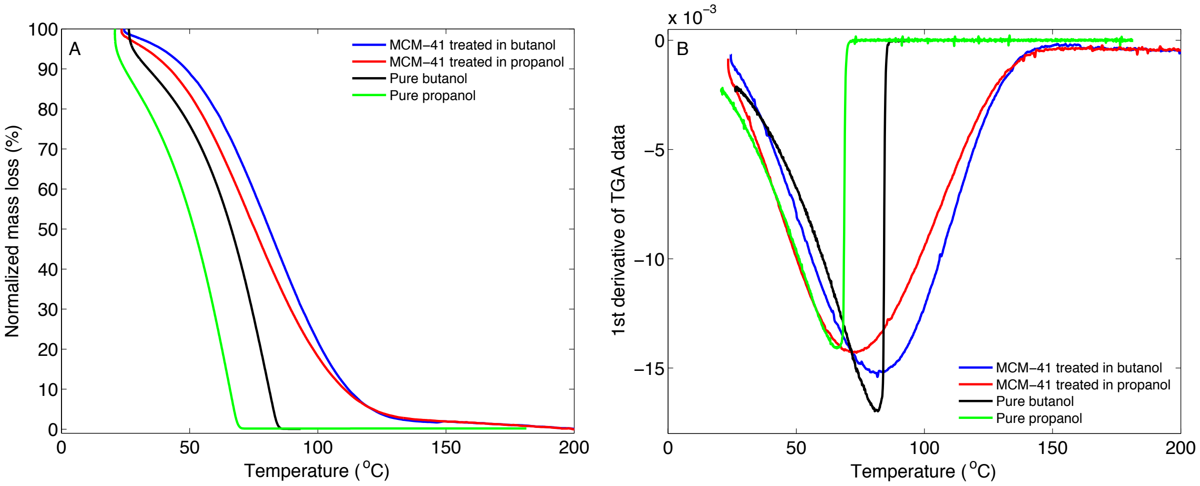

Figure S3. (A) TGA data of MCM-41 treated in propanol or butanol and the corresponding pure alcohols. In this figure the normalized mass loss (%) is defined as m/m_{initial} for the pure alcohols and $(m-m_{T=200°C})/(m_{\text{initial}}-m_{T=200°C})$ for the MCM-41 samples to allow for comparison. (B) First derivative of the normalized mass curves in (A) as a function of temperature.

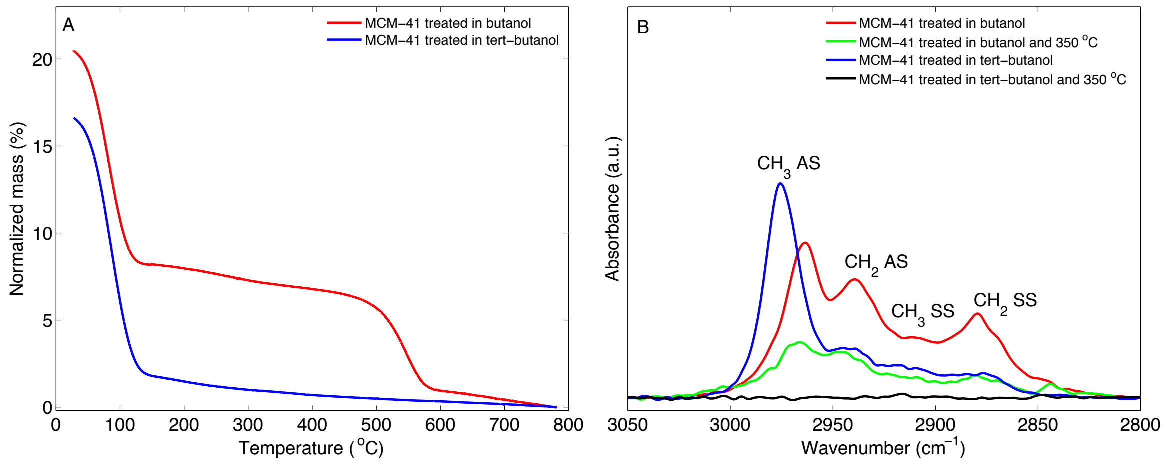

Figure S4. (A) TGA data of MCM-41 treated in normal butanol and tert-butanol. The TGA curve from the sample treated in tert-butanol only shows a mass loss step at $T_{1,TGA}$=50-150°C, while the mass loss step at $T_{2,TGA}$=400-600°C is not present. In other words, tert-butanol is only physically adsorbed, and not chemically attached, to the MCM-41 material. (B) FTIR data on MCM-41 treated in normal butanol and tert-butanol, before and after heating the samples to 350°C. The FTIR trace from the sample treated in tert-butanol does not show any signs of C-H vibrations after heating to 350°C. The intensity of all spectra are normalized with respect to the most prominent absorption band between 950-1250 (not shown here), which is assigned to antisymmetric stretching of siloxane.

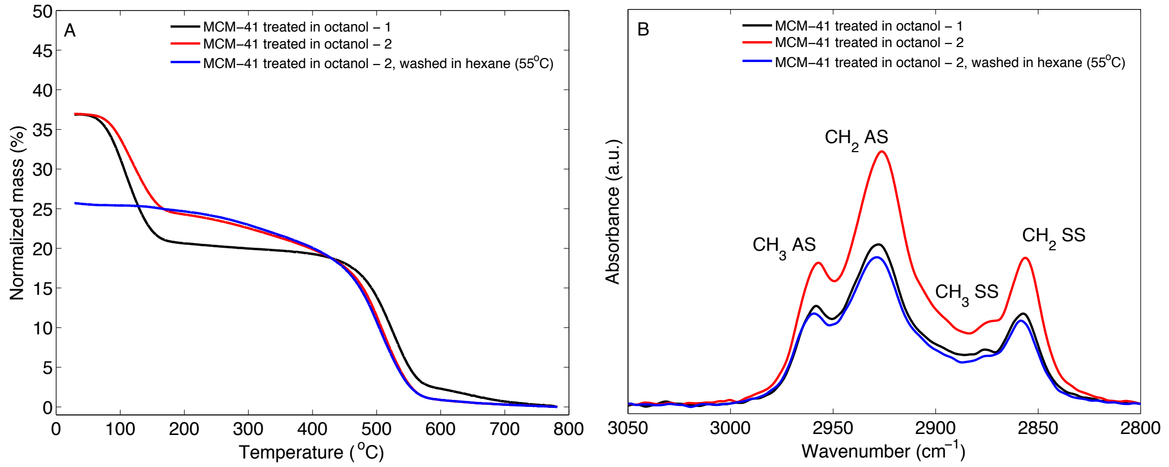

Figure S5. (A) TGA data of MCM-41 treated in octanol before and after washing in hexane at 55°C (these control experiments were performed with a 2nd batch of MCM-41 treated in octanol, the batch number is indicated by number 1 or 2). The TGA curve from the sample washed in hexane does not have any mass loss step at $T_{1,TGA}$=50-150°C, while the mass loss step at $T_{1,TGA}$=400-600°C is still present even after rigorous washing in hexane at elevated temperature. (B) FTIR data on the corresponding samples as shown in (A). The FTIR trace from the sample washed in hexane has clear peaks from C-H vibrations, which are similar to the sample before washing. The intensity of the peaks is however lower indicating that the fraction of physically adsorbed molecules is absent. The washing procedure was as follows. First the octanol treated MCM-41 was put in 2 ml hexane at 55°C for 1h with mixing. After centrifugation the supernatant was removed and 2 ml of fresh hexane was added. This washing step was repeated 4 times in total. Finally the sample was dried at reduced pressure before investigated by TGA and FTIR. In both (A) and (B) the results from the 1st batch of MCM-41 treated in octanol are included for comparison (same data as shown in Figs. 2 and 3 in the main article).

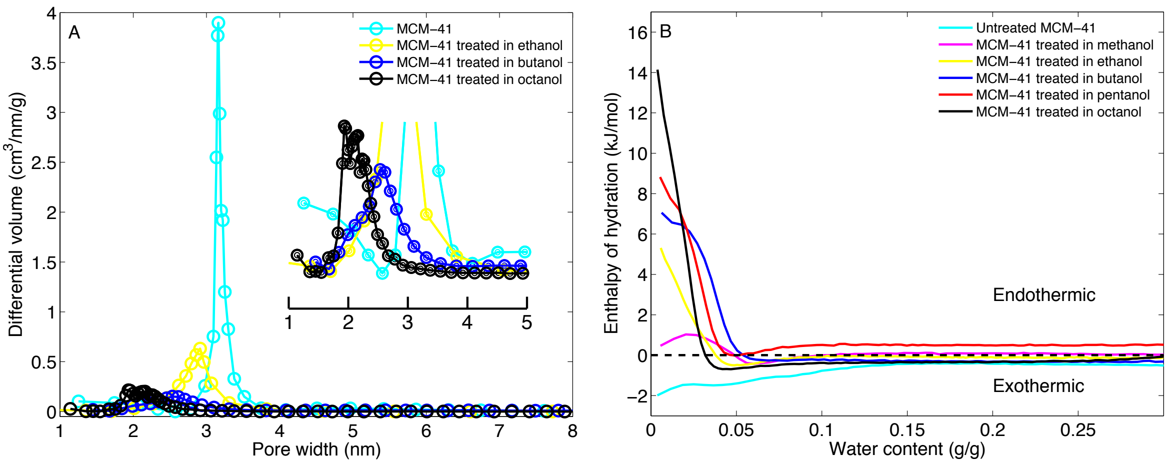

Figure S6. (A) Pore size distributions of untreated MCM-41 and alcohol treated MCM-41 samples based on hexane sorption isotherms (see Fig. 5B in the main article). The pore width was determined to 3.2 nm for untreated MCM-41, and 2.9, 2.5, and 2.0 nm for MCM-41 treated in ethanol, butanol, and octanol, respectively (see inset in (A) for close up). The BJH equation was used for these calculations with t=5 Å and the contact angle θ=0° for all samples. (B) Hydration enthalpy as a function of water content from the corresponding sorption isotherms of MCM-41 and alcohol treated MCM-41 samples presented in Fig. 5 in the main article. The hydration enthalpies at relatively high water contents, above the capillary condensation regime, are omitted due low sensitivity in this region. It should be noted that the alcohol treated MCM-41 samples were dried in vacuum with molecular sieves for 12 h minimum before the sorption calorimetry experiments to avoid excess of alcohols in the mesopores.

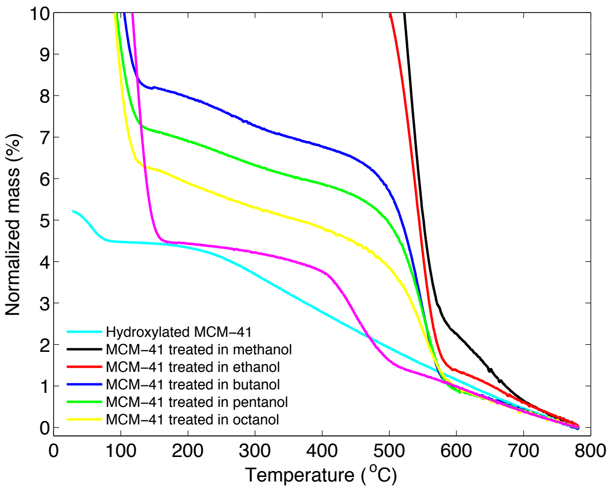

Figure S7. Comparison of TGA curves from hydroxylated MCM-41 and alcohol treated MCM-41. The TGA curve from the hydroxylated MCM-41 sample shows similar slope as the alcohol treated samples in the temperature interval where no clear mass loss step is observed, i.e. between approximately 200-400°C and 700-800°C. This indicates that the alcohol treated samples contain a significant number of hydroxyl groups that are continuously vaporized between approximately 200-400°C and 700-800°C during the TGA experiments.

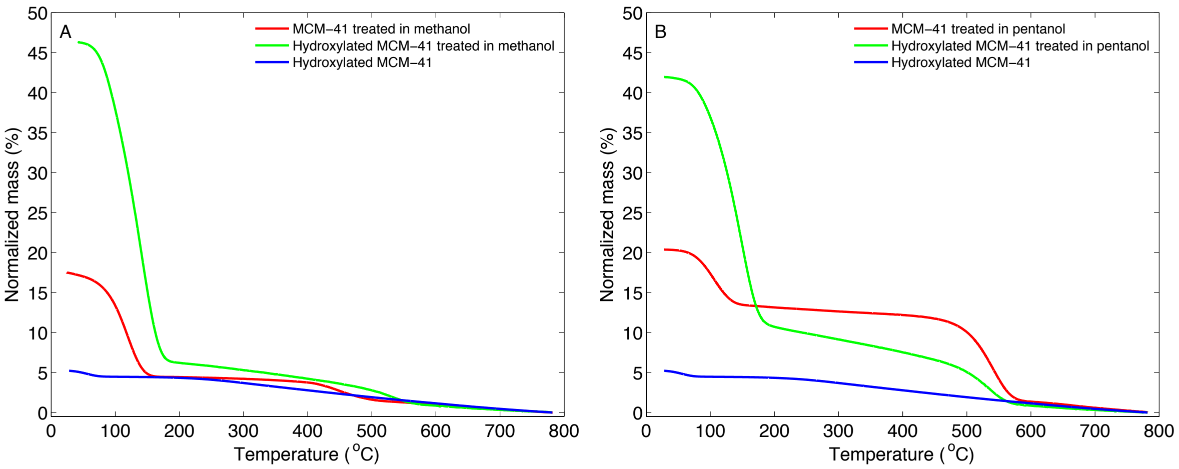

Figure S8. Comparison of MCM-41 and hydroxylated MCM-41 treated in alcohol. (A) Samples treated in methanol. (B) Samples treated in pentanol. The TGA curve from hydroxylated MCM-41 (without alcohol treatment) is included for comparison. The slope of the TGA curves between approximately 200-400°C is steeper for the hydroxylated samples, as compared to the other samples, which indicates that the mass loss in this region reflects both losses of silanol and alkoxy groups. Due to that the second mass loss step, between 400-600°C, is less defined for the hydroxylated samples it is difficult to determine the number of covalently bound alkoxy groups for these samples.
